# Comparison of the efficacy of platelet-rich plasma with topical minoxidil in treating patients with androgenetic alopecia: a systematic review of clinical trials

**DOI:** 10.1093/skinhd/vzaf040

**Published:** 2025-07-23

**Authors:** Areesha Abid, Faizan Fazal, Haris Mumtaz, Hafsa Arshad Azam Raja, Bilal Haider Malik

**Affiliations:** Department of Medicine, Rawalpindi Medical University, Rawalpindi, Pakistan; Department of Medicine, Rawalpindi Medical University, Rawalpindi, Pakistan; Department of Medicine, Rawalpindi Medical University, Rawalpindi, Pakistan; Department of Medicine, Rawalpindi Medical University, Rawalpindi, Pakistan; ST6 Dermatology, Department of Dermatology, Betsi Cadwaladr University Health Board, Wrexham Maelor Hospital, Wrexham, UK

## Abstract

Androgenetic alopecia (AGA) is a very common cause of noncicatricial alopecia, which negatively affects a person’s wellbeing. Although Food and Drug Administration (FDA)-approved drugs such as topical minoxidil result in an apparent improvement in this hair condition in a period of 4–6 months and have been used commonly as the first-line treatment of choice, another treatment modality that has gained popularity over the years is platelet-rich plasma (PRP) therapy. PRP is minimally invasive but much more cost-effective than restoration surgery. The FDA has not approved PRP as a treatment modality for AGA. We systematically reviewed the existing literature from Embase, Web of Science, CENTRAL and PubMed from inception to 2024, and included six clinical trials that compared these two commonly practised dermatological therapies for the treatment of AGA. Most studies used global photographic assessment of hair changes based on the investigator’s examination, which demonstrated statistically significant changes in hair density, terminal hair count and hair pull test. A few studies used subjective quantitative measures of hair parameters, such as patient satisfaction scores and improvement in hair quality. Topical minoxidil showed more improvement in terminal hair count and proportion of anagen hair. PRP showed more improvement in hair density and a negative hair pull test. All of the selected studies suggested that the efficacy of PRP is nearly comparable to that of topical minoxidil, with minimal adverse effects on long-term follow-up. Thus, PRP is a valuable treatment option either adjuvant to topical minoxidil or as a second-line treatment option for AGA.

## Graphical Abstract

**Graphical Abstract:**
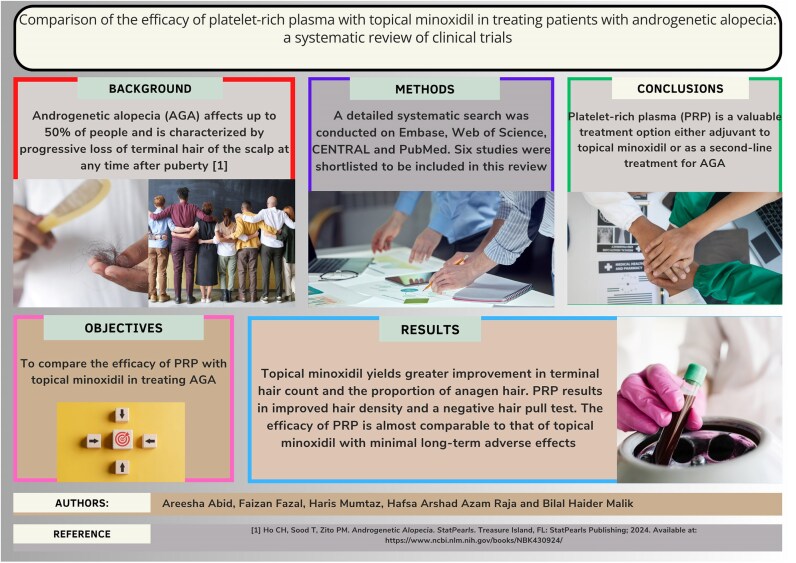

## Teaser

All of the included studies in this review article suggest that the efficacy of PRP is nearly comparable to that of topical minoxidil, with minimal adverse effects on long-term follow-up. Thus, PRP is a valuable treatment option either adjuvant to topical minoxidil or as a second-line treatment option for AGA.

What is already known about this topic?

- Topical minoxidil is a Food and Drug Administration-approved first-line treatment for androgenetic alopecia (AGA).
- Platelet-rich plasma (PRP) is an emerging and alternative treatment for AGA, but its efficacy is not completely known.

What does this study add?

- PRP’s efficacy is comparable to topical minoxidil.
- PRP can be used as an alternative therapy or as an adjunct to topical minoxidil in treating AGA.

Androgenetic alopecia (AGA) negatively affects a person’s mental wellbeing. It results in feelings of anxiousness, helplessness and diminished self-esteem.^1^ Additionally, in some parts of the world, AGA may be affiliated with cultural stigmas.^2^

In addition to lifestyle improvement and haircare practices, the first-line line treatment of AGA is finasteride and topical minoxidil (TM), as these result in apparent improvement in a period of 4–6 months.^3^ Another treatment modality that has gained popularity over the years is platelet-rich plasma (PRP), as it is considered to be minimally invasive for patients with AGA.^4^ Minoxidil was initially used for hypertension but was repurposed for hair loss after the observation that patients experienced a significant increase in hair growth as a side effect of this medicine. It is theorized that minoxidil causes scalp blood vessel dilation, increasing nutrient delivery to the hair follicles.^3^ Minoxidil achieves this result by affecting the potassium channels in the vascular smooth muscles in scalp vessels. Activation of these channels relaxes the smooth muscles, which causes dilatation of such vessels.^5^

It is commonly known that during wound healing, active platelets release growth factors and cytokines through alpha granules. These growth factors, when the platelets are injected into the scalp, promote hair growth.^4^ PRP is relatively safe but may also cause side effects such as temporary pain and oedema. However, no major side effects have been observed.^6^

The above-mentioned studies show that the effects and side effects of these treatment modalities have been extensively studied. However, the need for uniform treatment still stands. Therefore, we conducted a systematic review and a meta-analysis to finally find a solution that might not only determine which option should be preferred by the physicians, but may pave the road for further studies in the future.

## Methodology

This study was performed in accordance with the Preferred Reporting Items for Systematic Reviews and Meta-Analyses (PRISMA) guidelines for writing and reporting systematic reviews and meta-analyses.

### Eligibility criteria

The research included patients who were diagnosed with AGA. We incorporated studies that evaluated TM with a PRP regimen using a double-arm, randomized controlled trial design. The studies included in the review had both men and women participants with AGA, the grade of which is classified according to the Hamilton-Norwood scale (men) and the Ludwig scale or Savin scale (women). Included studies were those that compared the use of TM with that of PRP for treating AGA. The papers did not include cohorts, single-arm trials, reported oral minoxidil or any other illness except AGA. Individuals who reported taking minoxidil orally or in any other way were not included. Only published works in the English language were included in the search parameters. To find more pertinent research, the lists of references in all qualifying articles were carefully examined.

### Search strategy used for the study

The publications found in this study were found by scanning many databases. We searched the scientific databases of Embase, Web of Science, CENTRAL and PubMed for all relevant papers that compared TM with PRP for the treatment of AGA. The following keywords and their corresponding medical subject headings terms were used: alopecia, androgenetic alopecia, platelet-rich plasma and minoxidil. All of the databases were searched from the time of their inception until July 2024.

### Screening of articles

Two investigators (H.M. and H.A.A.R.) independently screened the titles and abstracts of each relevant study for inclusion. The full text of potentially relevant articles was reviewed by two reviewers (H.A.A.R. and H.M.) independently. Discrepancies were resolved by discussion with the entire group of authors.

### Extraction of data from the included studies

Two authors (A.A. and F.F.) independently extracted the data using a standardized data extraction sheet and established inclusion criteria in a rigorous and transparent manner. The authors (A.A. and F.F.) discussed and came to a consensus on any disagreements or inconsistencies during the data extraction process; a third author (B.H.M.) was consulted if needed. Two investigators independently screened each citation for inclusion. To ascertain their eligibility, two reviewers (F.F. and A.A.) independently examined the full-text publications of possibly pertinent studies. The following data were extracted from the included studies: study name, year, URL/DOI, study type, sample size, inclusion and exclusion criteria, intervention, comparator, follow-up, outcome, adverse events, potentially poolable outcome, the baseline characteristics included; study ID, year of publication, study design, outcomes assessed and results.

### Quality assessment of the included studies

To examine the bias risk in the included studies, a quality evaluation was conducted. Two authors independently evaluated bias using the Cochrane Risk of Bias (RoB 2.0) technique for systematic intervention reviews. Several bias domains, such as those pertaining to randomization, selective reporting, missing outcomes data, intervention adherence and outcome measurement, were taken into account in this evaluation.

## Results

**Figure 1:**
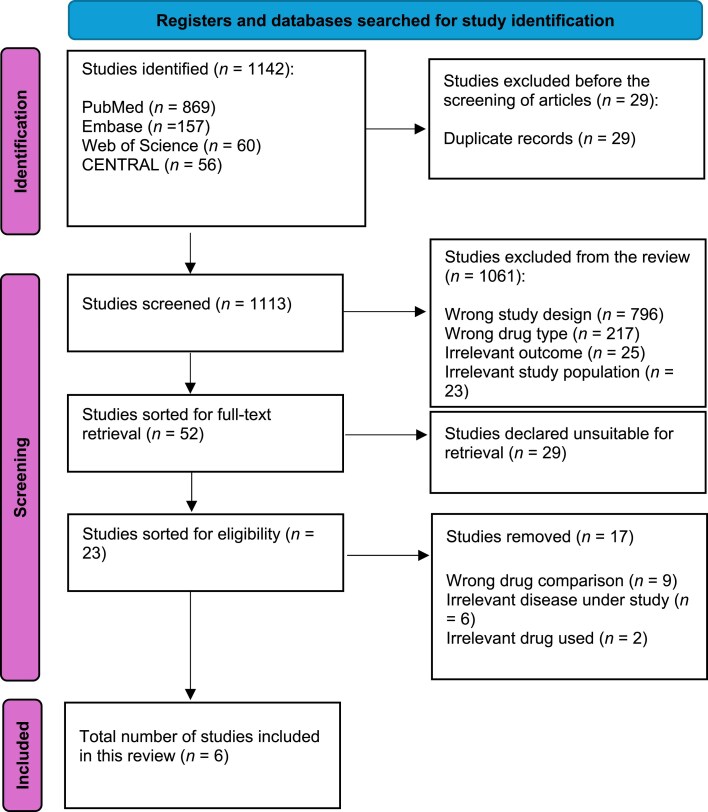
PRISMA flowchart regarding scheme of included studies.

Out of a total of 1142 studies, only 6 were considered to meet our inclusion criteria and were included in this systematic review. The whole scheme of study selection is shown in Figure 1.

The main outcomes in all of the included studies were global photographic assessment of hair parameters, including changes in hair density, changes in hair count, hair pull test and patient satisfaction. However, the primary and secondary outcomes in each study differed. In one of the included studies, hair density was calculated by marking 1 cm^2^ patches on five different spots within the affected area of the scalp, then counting the hairs in each spot. In another study that reported this outcome, improvement was reported by the use of dermoscopic pictures taken at a fixed site. Other included studies that also reported the same outcome chose investigator-rated change in hair density based on re-grading of the patient’s alopecia and global photography for baseline and post-treatment photographs as reviewed by the blinded evaluator. In the hair pull test, about 60 hairs were tugged between the thumb and the index and middle fingers. The hairs were then gently but firmly pulled. More than 4–6 hairs were taken as a ‘positive’ hair pull test, while fewer than 4–6 hairs were taken as a ‘negative’ hair pull test in most of the included studies that reported this outcome. Patient satisfaction outcome in our included studies was determined by either the use of a validated self-administered questionnaire or a visual analogue score.

Adverse events, such as mild headaches, dryness, scalp pruritus, increased facial hair, itching, scaliness and local scalp irritation, were also reported in the included studies.

All studies compared and studied the effect of TM vs. PRP in treating AGA. Detailed characteristics of the study population and the relevant treatment administered are shown in Table 1.

**Table 1:** Details of the study groups and the treatment given in the six studies included in the systematic review

Details	Balasundaram *et al.*, 2023^15^	Afzal *et al.*, 2024^7^	Asim *et al.*, 2023^10^	Singh *et al*., 2020^8^	Farid and Abdelmaksoud, 2016^9^	Verma *et al.*, 2019^11^
Country	India	Pakistan	Pakistan	India	Egypt	India
Study design	RCT	RCT	RCT	RCT	RCT	RCT
Total number of patients	64	70	72	36	40	40
Age of participants (years)	20–50	18–60	20–60	18–60	N/A	20–49
Age of patients (years), mean (SD)	27.09 (3.13) in minoxidil group, 26.61 (3.05) in PRP group	28 (6.8) in minoxidil group, 26.9 (5.9) in PRP group	26.44 (6.79) in minoxidil group, 28.63 (6.15) in PRP group	26.9 (3.9) in minoxidil group, 26.5 (4.5) in PRP group	26.1 (5.41) in minoxidil group, 29.6 (8.38) in PRP group	25.07 (4.5) in minoxidil group, 25.7 (3.8) in PRP group
Men/women included	Men	Men and women	Men and women	Men	Men and women	Men
Disease studied	AGA	AGA	AGA	AGA	AGA	AGA
Grade according to modified Hamilton-Norwood classification	3 and 4	≥2 and ≤5	≥1 and ≤7	≥2 and ≤5	≥1 and ≤6	≥1 and ≤5
Number of patients in the PRP group	25	35	36	17	20	16
Method of PRP administration	Injected with a 0.1–0.2 mL dose at a depth of 3–4 mm	Injected with a 0.1–0.2 mL dose at a depth of 3–4 mm	Injected with a 0.1–0.2 mL dose at a depth of 3–4 mm	Injected intradermally at a distance of 1 cm and in doses of 0.05–0.1 mL cm^–2^	1 mL of scalp mesotherapy and 1 mL of microneedling	Injected with a 0.1–0.2 mL dose at a depth of 3–4 mm
Duration of PRP treatment	Given monthly for a period of 3 months	Given monthly for a period of 6 months	Given monthly for a period of 3 months	Given monthly for a period of 3 months	Given monthly for a period of 6 months	Given monthly for a period of 4 months
Number of patients in the TM group	26	35	36	19	20	14
Dose of TM given	5% in a 1 mL dose that needed to be applied twice a day	5% in a 1 mL dose that needed to be applied twice a day	5% in a 1 mL dose that needed to be applied twice a day	5% in a 1 mL dose that needed to be applied twice a day	5% in a 1 mL dose that needed to be applied twice a day	5% in a 1 mL dose that needed to be applied twice a day
Primary outcome	Global photographic assessment, total hair density, change in hair count, proportion of anagen hairs	Global photography	Hair pull test	Global photographic assessment, hair density	Hair density, patient satisfaction, and rate of hair shedding	Global photography, hair pull test, hair growth, patient satisfaction score
Secondary outcome	Adverse events, patient satisfaction	Hair pull test, Patient satisfaction score	Hair density	Patient satisfaction	Adverse effects, tolerability	N/A

AGA, androgenetic alopecia; N/A, not available; PRP, platelet-rich-plasma; RCT, randomized controlled trial; TM, topical minoxidil.

Each of the included studies had primary and secondary outcomes to assess and compare the effectiveness of TM and PRP. Although TM is an approved drug used for AGA, almost all studies included in this systematic review showed that PRP can have a significant impact on improving outcomes in patients with AGA: 91%, 100% and 45% of the participants showed improved patient satisfaction in the PRP therapy group, respectively, in studies conducted by Afzal *et al*. (2024),^7^ Singh *et al*. (2020),^8^ and Farid and Abdelmaksoud (2016);^9^ Asim *et al*. (2023),^10^ showed that PRP was more effective than TM in treating AGA. Farid and Abdelmaksoud (2016)^9^ and Verma *et al*. (2019)^11^ concluded that PRP could be an effective second-line drug for AGA after TM. Singh *et al*. (2020)^8^ came to the opinion that using a combination of PRP and TM can be more effective than using either PRP or TM alone, as they observed in that the mean change of hair density from baseline was 49.45 in the PRP group, while the mean change from baseline was 33.30 in the TM group. Detailed descriptions of primary and secondary outcomes of the six included studies are shown in Table 2.

**Table 2:** Details of primary and secondary outcomes of the included studies

Outcome	Balasundaram *et al.*, 2023^15^	Afzal *et al.*, 2024^7^	Asim *et al.*, 2023^10^	Singh *et al*., 2020^8^	Farid and Abdelmaksoud, 2016^9^	Verma *et al*.,^11^ 2019
Hair density in the PRP group, mean (SD)	The mean change from baseline was 8.18	N/A	N/A	The mean change from baseline was 49.45 (5.82)	N/A	N/A
Hair density in the TM group, mean (SD)	The mean change from baseline was 7.12	N/A	N/A	The mean change from baseline was 33.30 (14.82)	N/A	N/A
Hair count in the PRP group, mean (SD)	The mean change from baseline was 17.68	N/A	N/A	N/A	The mean change from baseline was 5.05 (27.95)	N/A
Hair count in the TM group, mean (SD)	The mean change from baseline was 25.27	N/A	N/A	N/A	The mean change from baseline was 16.05 (11.83)	N/A
Global photographic assessment in PRP group	38% of the patients responded to treatment	N/A	N/A	N/A	N/A	N/A
Global photographic assessment in TM group	56% of the patients responded to treatment	N/A	N/A	N/A	N/A	N/A
PS in PRP group	median PS for hair texture was better for minoxidil than PRP	91.4% PS	N/A	Improvement noted by 19 (100%)	Improvement 9 (45%)	6.56 (1.09)
PS in TM group	N/A	Improvement noted by 54.2% of participants	N/A	Improvement noted by 94.1% of participants	Improvement noted by 65% of participants	N/A
Negative hair pull test in the PRP group	N/A	27 (77%)	33 (91.7%)	N/A	N/A	12 (75%)
Negative hair pull test in the TM group	N/A	14 (40%)	25 (69.4%)	N/A	N/A	6 (42.8%)
The proportion of anagen hairs in the PRP group	At 12 weeks were 0.87 (0.04)	N/A	N/A	N/A	N/A	N/A
The proportion of anagen hairs in the TM group	At 12 weeks were 0.89 (0.04)	N/A	N/A	N/A	N/A	N/A
Adverse effects in the PRP group	53% in the PRP arm; pain was the most common side effect	N/A	N/A	Instant pain (100%)	100% pain at the injection site, 30% headache	N/A
Adverse effects in the TM group	Dryness, pruritus on the scalp, and minor headaches were typical adverse effects	N/A	N/A	Instant pain (100%), scalp pruritis (15%), increased facial hair (5%)	30% local scalp irritation, 25% itching and scaliness, 5% dry scaly flakes	N/A
Conclusion	PRP works well for treating men with intermediate grades of AGA	For the treatment of AGA, TM medication in conjunction with PRP therapy can be quite beneficial	When treating AGA, PRP therapy shows better efficacy than minoxidil	Topical minoxidil plus PRP works better together than PRP and TM alone	PRP combined with microneedling may be a useful second-line treatment for individuals who are not suitable for minoxidil or who are intolerant to it	For the treatment of AGA, TM medication may be usefully substituted with PRP therapy

AGA, androgenetic alopecia; N/A, not available; PRP, platelet-rich-plasma; PS, patient satisfaction TM; topical minoxidil.

**Figure 2:**
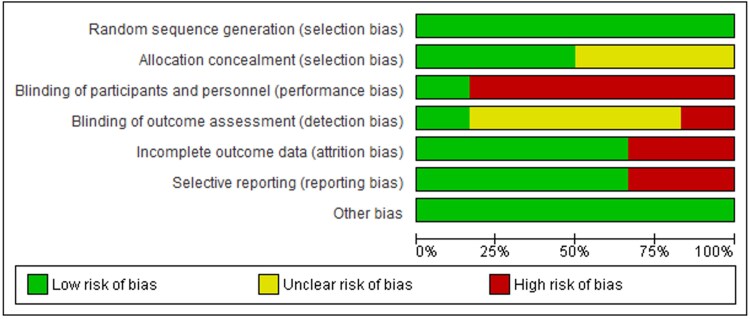
Risk of bias graph.

Seven different risk of bias components were studied in each of the six included studies. Each of the six studies was evaluated by the authors of this systematic review for potential risk of bias. All six studies had random sequence generation used in their studies. Blinding of participants and personnel was concluded to be of high risk of bias in the majority of included studies. Figure 2 shows the graphical representation of the risk of bias in the included studies.

**Figure 3:**
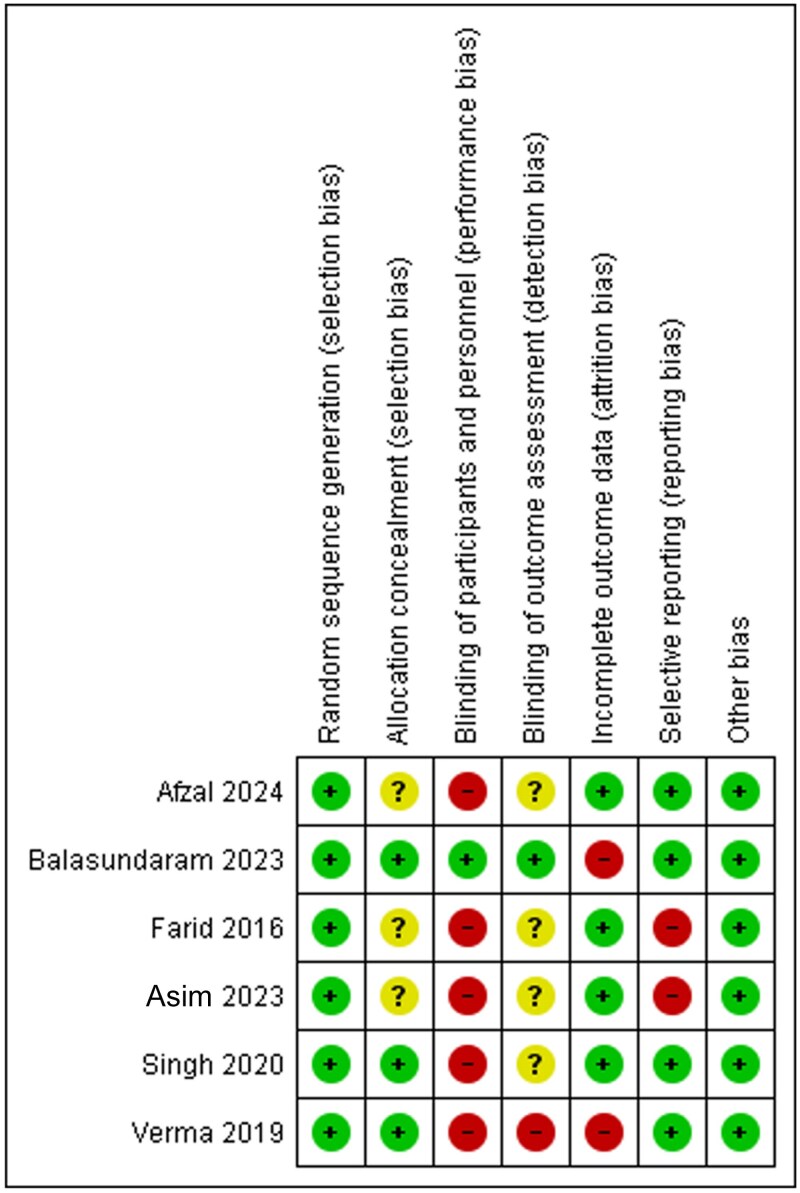
Risk of bias summary.

Figure 3 shows the summary of the risk of bias in the form of a pictorial representation of all seven risk of bias components in each of the included studies.

## Discussion

The potential of PRP to stimulate hair growth and reverse the symptoms of AGA has been thoroughly investigated in many clinical trials published in the literature.^12–14^ Our selected studies evaluated the response of treatment for AGA based on one or more of standard criteria which included (i) change in hair density, (ii) increase in hair count, (iii) hair pull test results and (iv) patient satisfaction scores, alongside global photographic assessment to demonstrate improvement and patient satisfaction scores as secondary outcomes.

Among the included studies, Balasundaram *et al*. (2023)^15^ and Singh *et al*. (2020),^8^ found PRP to be superior to TM in clinical efficacy by the primary outcome, which was changes in hair density. Balasundaram *et al*. (2023)^15^ reported greater increases in hair density with PRP, while results for other secondary outcomes, such as terminal hair count, portion of anagen hair and patient satisfaction for hair texture, were profoundly efficacious for TM. Global photography was used as the diagnostic tool for evaluation. It revealed that all male participants in the study responded better to TM for secondary outcomes (56% vs. 38% at week 24) than to PRP, indicating that PRP is equally effective as minoxidil in treating moderate grades of AGA in men. Singh *et al*. (2020)^8^ discovered that male participants who were split into four groups experienced higher gains in hair density outcomes: hair density increased in the PRP plus minoxidil group, PRP alone and minoxidil alone groups (in decreasing order), whereas the placebo-controlled group showed a reduction in hair density.

In terms of the outcome of changes in hair count, Balasundaram *et al*. (2023)^15^ and Farid and Abdelmaksoud (2016)^9^ both found TM to be superior to PRP. Farid and Abdelmaksoud (2016)^9^ found that TM increased the number of terminal hairs after performing a nonvellus hair count in a target area after 12 and 28 weeks of treatment, achieving a change of 16.05 from baseline as compared with only 5.05 from baseline for PRP. This also suggested that PRP can be a useful second-line treatment for patients who are intolerant to or unsuitable for minoxidil.

Among our other included studies, Afzal *et al*. (2024),^7^ Asim *et al*. (2023)^10^ and Verma *et al*. (2019)^11^ conducted similar trials on both the male and female populations of South East Asia diagnosed with AGA grade >1. These three studies reported comparative efficacy of both treatments with negative hair pull test as a primary outcome and found PRP to be superior to TM. In all of the studies, the greatest number of participants achieved a negative hair pull test with PRP in comparison with TM in 6 months, 3 months and 4 months, respectively. Afzal *et al.*^7^ showed that PRP was effective in >70% of participants and 5% TM in <50% of participants in their study. Similarly, Asim *et al*.^10^ found that the PRP therapy group had a negative hair pull rate of 91.7%, higher than the minoxidil-treated group’s rate of 69.4%. In a related trial with 40 participants, Verma *et al*.^11^ showed with the similar outcome that PRP therapy can be a useful substitute for TM therapy in the management of AGA.

Among our reviewed studies that evaluated patient satisfaction as a main outcome, Afzal *et al.* (2024)^7^ and Singh *et al.* (2020)^8^ reported the majority of patient satisfaction results in favour of PRP as superior to TM, while Farid and Abdelmaksoud (2016)^9^ reported a similar outcome in favour of TM as superior in comparison with PRP.

In previous literature, Georgescu *et al*. (2022), who conducted a similar study, were able to retrieve quantitative estimates of changes in hair density (from an initial value of 141.9 hairs cm^–2^ to 177.5 hairs cm^–2^ at the last evaluation) obtained with reliable standardized methods in 14 out of 15 clinical studies on PRP for AGA.^16^ A recent meta-analyses also showed that PRP significantly increased hair density in subjects with AGA with a more pronounced effect in men.^17^

Topical minoxidil is very effective in promoting total and nonvellus hair growth.^18^ The significant increase in hair count with TM was also proved effective in a placebo-controlled multicentric study in the USA. Hence, when looking for significant increases in hair count, TM seems to be of more clinical value.^19–22^ However, more studies are needed to further demonstrate its efficacy in this regard.

Yepuri and Venkataram (2021) enlisted 60 patients with AGA who did not respond to FDA-approved finasteride or minoxidil and then used microneedling to assess each patient’s reaction to PRP. In terms of hair growth and thickness, more than 80% of the trial participants showed at least a 40% improvement over their baselines, and of them, 66% of patients who underwent at least four PRP sessions had good to excellent results on subjective assessment. This shows PRP can be a beneficial treatment option for those who fail to respond to TM.^23^

In contrast to the above results, studies conducted in Austria (Gressenberger *et al*.) and western India (Agarwal *et al*.) reported insignificant improvement in hair density with PRP.^24,25^ Factors that could explain variations in the outcomes reported from different trials include the time and techniques for evaluating the clinical outcomes, the properties of PRP and the frequency of treatment administration.^25^ A recent systematic review conducted by a student author at the University of California found that more patients who underwent PRP treatment for AGA achieved more positive outcomes in the hair pull test and comparatively more patient satisfaction than those who received TM alone.^26^ Tawfik and Osman (2017) also reported a negative hair pull test in PRP-injected areas in more than 80% of patients, whereas in placebo areas, the hair pull test remained positive at the 6-month follow-up duration.^27^

Global photography is also used to evaluate responses to the treatment in patients with AGA. This standard method has also been used by many clinical studies in the literature. Tawfik and Osman used global pictures to show a significant improvement in hair volume and quality using PRP.^27^ It was also used to assess the patient’s hair loss compared with baseline in a study conducted to evaluate the clinical efficacy of 5% TM in comparison with placebo in experimental studies in the USA.^22^

In all clinical trials, although PRP caused some brief but manageable pain during therapy, overall, it was a relatively safe intervention.^6,28^ The experimental studies selected in our review reported scalp pruritus, irritation and dryness. One of them reported an increase in facial hair with the use of TM for the treatment of AGA. These effects of TM are common and are documented in the literature.^29^

There has not been much research in the literature comparing the effectiveness of PRP therapy vs. minoxidil therapy, despite many studies documenting their individual efficacies in the past.^13^ In this regard, our review provides a powerful insight, where the comparison is made between two routinely performed dermatological and aesthetic procedures in patients with AGA. However, the limited number of studies available highlights the need for more clinical evidence to prove the efficacy of PRP over TM.

The existing literature on PRP does have some problems, as it reports different PRP techniques and lacks consistent agreement that would allow for an unbiased analytical comparison. The included studies have a limit to their statistical reproducibility by use of subjective quantitative assessment of hair parameters rather than standard phototrichographic assessment, which in one study was preferred owing to the treated person’s inconvenience. The way that PRP preparation techniques are reported in clinical trials varies widely, and the majority of them do not provide enough information to allow for process replication. Furthermore, the current reporting of PRP preparation and composition makes it impossible to compare the PRP products that are administered to patients. A comprehensive, precise and step-by-step description of the PRP preparation process is required for repeatability and research comparability. The loss to follow-up of a few patients in the included studies of the review was due to withdrawal of consent as a result of dissatisfaction, and adverse effects such as pain and discomfort.

A meta-analysis could not be performed due to non­uniformity of the outcomes metrics among all six included randomized controlled trials, resulting in the authors of this study not being able to analyse any of the outcomes, which is a limitation to the review; however, the review is unique in the sense that it covers the assessment of maximum number of clinically observable hair parameters through validated techniques and report the clinically efficacy of the desired intervention whenever chosen by the investigators anywhere in the world. Although the authors searched vigorously through all the studies to find the relevant studies to be included, more data are needed to make a more generalized comment on the debated topic.

The clinical efficacy of PRP is nearly comparable to 5% TM. Whereas TM shows more improvement in one or more hair parameters, such as terminal hair count and proportion of anagen hair, in different studies, PRP proves more efficacious in others, specifically the hair density and negative hair pull test, with more studies giving higher patient satisfaction in favour of PRP. Hence, PRP is a valuable treatment option either adjuvant to minoxidil or as a second-line treatment of choice in the treatment of AGA.

## Data Availability

The data relevant and related to this study are available in the Zonedo, at https://zenodo.org/records/13356293 and can be accessed with doi.org/10.5281/zenodo.13356293.^30^
